# Association between GRIN2B polymorphism and Parkinson’s disease risk, age at onset, and progression in Southern China

**DOI:** 10.3389/fneur.2024.1459576

**Published:** 2024-12-20

**Authors:** Can Cui, Hongxia Li, Yiwen Bao, Yingying Han, Hongxiang Yu, Huan Song, Bei Zhang

**Affiliations:** ^1^Department of Neurology, Shanghai East Hospital, School of Medicine, Tongji University, Shanghai, China; ^2^Department of Digestive Diseases, Huashan Hospital, Fudan University, Shanghai, China

**Keywords:** GRIN2B, Parkinson’s disease, motor progression, LOPD, rigidity, axial impairment

## Abstract

**Background and objectives:**

The role of N-methyl-D-aspartate receptor 2B (GRIN2B) single nucleotide polymorphisms (SNPs) in influencing the risk and progression of Parkinson’s disease (PD) is still unclear. This study aimed to assess the impact of GRIN2B genotype status on PD susceptibility and symptom progression.

**Methods:**

We enrolled 165 individuals with sporadic PD and 154 healthy controls, all of whom had comprehensive clinical data available at the start and during follow-up. We used chi-squared (χ^2^) analysis to compare the allele and genotype frequency distributions between the patient and control groups. Linear mixed-effect models were employed to investigate the link between the GRIN2B genotype and the progression of motor and cognitive symptoms.

**Results:**

The prevalence of the GG + GT genotype and G allele was higher in patients compared to controls (*p* = 0.032 and *p* = 0.001, respectively). Subgroup analysis revealed that the GG + GT genotype and G allele were significantly more frequently observed in late-onset PD (LOPD) patients compared to early-onset PD (EOPD) patients (*p* = 0.014 and *p* = 0.035, respectively). Notably, individuals with the GG + GT genotype exhibited an estimated annual progression rate of 6.10 points on the Unified Parkinson’s Disease Rating Scale (UPDRS), which is significantly higher than that of the TT genotype carriers. Furthermore, the GG + GT carriers showed a markedly rapid progression in rigidity. In addition, the GG + GT carriers demonstrated significantly faster progression rates in rigidity (1.83 points/year) and axial impairment (1.2 points/year) compared to the TT carriers. Notably, the GG genotype carriers exhibited a more rapid decline in recall function.

**Conclusion:**

The GRIN2B rs219882 G allele is associated with increased PD susceptibility, particularly in LOPD. The carriers of the GG + GT genotype exhibited more rapid motor symptom progression, with a pronounced impact on rigidity and axial impairment.

## 1 Introduction

Parkinson’s disease (PD) is the most prevalent movement disorder, affecting an estimated 6 million individuals globally (1). The clinical manifestations of PD are multifaceted, encompassing both motor symptoms—such as tremors, rigidity, postural gait abnormalities, and bradykinesia—and non-motor symptoms (NMSs), including cognitive impairment (2). The etiology of PD remains complex, with genetic variations emerging as pivotal contributors to its pathogenesis (3). In recent years, a growing body of evidence has implicated numerous novel single nucleotide polymorphisms (SNPs) in PD, highlighting the essential role of these genetic variants in the molecular mechanisms that increase susceptibility to the disease (4).

An increasing body of research suggests that glutamate-mediated excitotoxicity plays a role in common neurological disorders, including PD (5, 6). The N-methyl-D-aspartate (NMDA) receptors, which are glutamate receptors, consist of NR1 (GRIN1) and NR2 (GRIN2) subtypes. The specific NR2 subunit (A, B, C, or D) determines the distinct physiological characteristics of NMDA receptors (7). Variation in the *GRIN2A* gene is the most common genetic cause of developmental and epileptic encephalopathy with spike-and-wave activation in sleep (DEE-SWAS) (8). N-methyl-D-aspartate receptor 2B (GRIN2B) is recognized as a key contributor to excitotoxicity-induced neuronal loss (9). Furthermore, GRIN2B dysfunction has been implicated in motor and cognitive deficits in *α*-synuclein-induced mouse models, and treatment with a GRIN2B inhibitor has been shown to rescue motor impairments in PD (10). However, the contribution of GRIN2B SNPs to PD and the impact of GRIN2B variations on PD development remain controversial. Some studies have indicated that GRIN2B SNPs reduce the risk of PD (11), while others have found that GRIN2B SNPs increase the risk of PD and the occurrence of impulse control behaviors (ICBs) in PD patients (12, 13). Several studies have suggested no correlation between GRIN2B SNPs and PD (13–15). The association between the GRIN2B genotype and the susceptibility of PD patients in China has not been well explored, and the influence of GRIN2B SNPs on PD progression has yet to be reported. Importantly, the impact of GRIN2B SNPs on the progression of PD has not been documented. Therefore, our study aimed to reveal the influence of the GRIN2B rs219882 genotype on PD susceptibility, age of onset, and the progression of motor and cognitive symptoms in a longitudinal cohort of individuals from southern China.

## 2 Materials and methods

### 2.1 Participants

**Figure 1:**
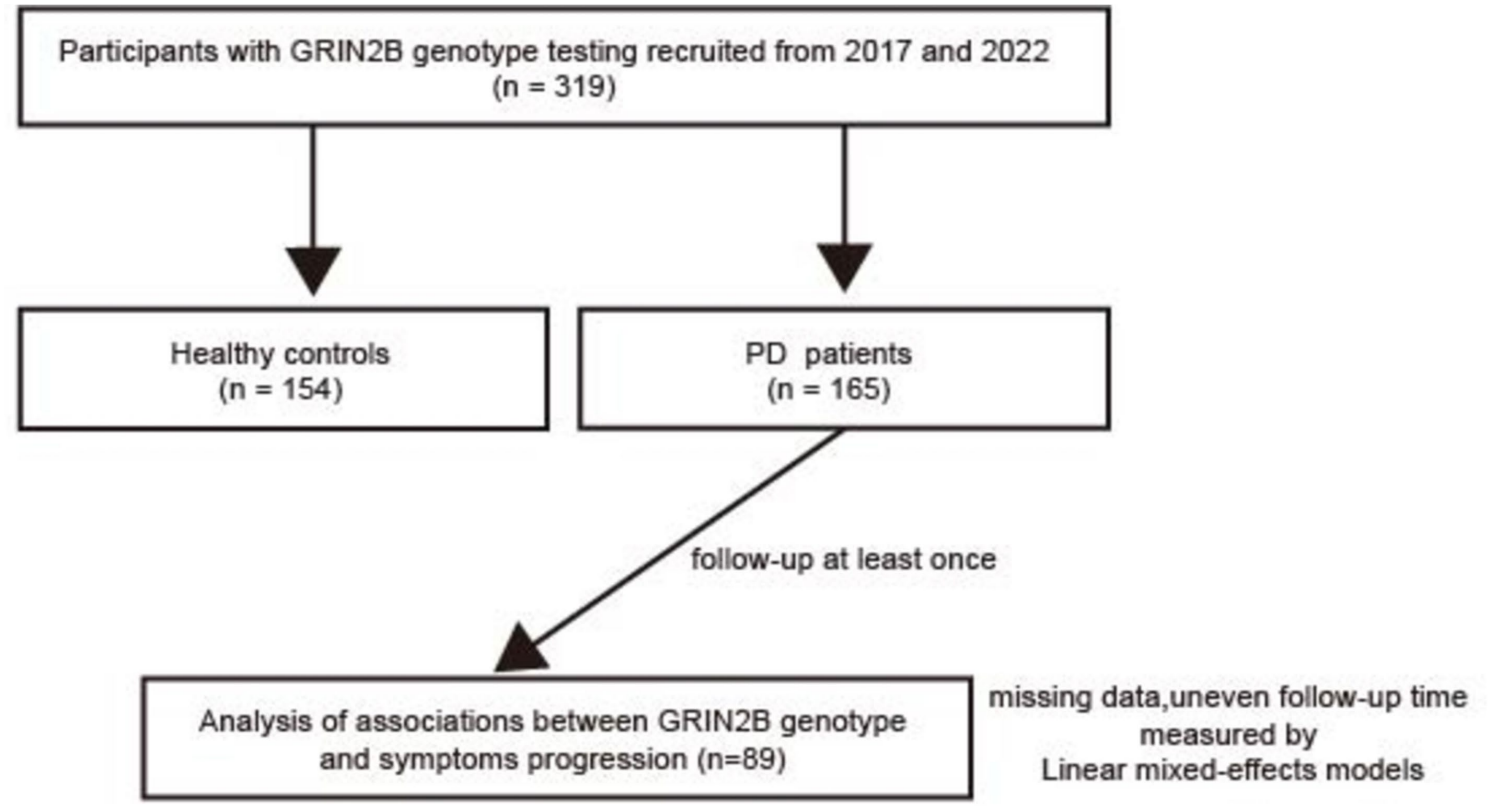
A flow chart of our study. PD, Parkinson’s disease; N-methyl-D-aspartate receptor 2B (GRIN2B).

This longitudinal study, conducted between 2017 and 2022, included a total of 165 individuals with sporadic PD from the Department of Neurology at Shanghai Dongfang Hospital (Figure 1). A control group comprising 154 healthy individuals, matched for sex and age, was recruited from the Zhoujiadu Community Health Service Center in the Shanghai Pudong New Area. All PD patients were diagnosed by neurologists who adhered to the clinical diagnostic criteria established by the UK Parkinson’s Disease Society Brain Bank (16). The inclusion criteria for the patients were as follows: (1) ability to provide informed consent; (2) diagnosis of idiopathic PD; (3) age between 30 and 90 years; and (4) ethnic Han Chinese descent. The exclusion criteria included the following: (1) parkinsonism attributable to other causes such as drugs, stroke, or toxins; (2) a familial history of PD; (3) the presence of other neurological disorders, including multiple system atrophy and PD dementia; and (4) a history of cerebrovascular diseases or other severe systemic conditions. The recruitment process and study protocols were approved by the Institutional Review Board (IRB) of Shanghai East Hospital (approval number: 2022135). All participants provided written informed consent in accordance with the ethical standards of the Declaration of Helsinki.

### 2.2 Clinical assessments

To evaluate the impact of GRIN2B SNPs on motor and cognitive progression in PD, a longitudinal follow-up study was conducted. Initially, 165 PD patients were enrolled, with 89 participants available and eligible for follow-up. The average follow-up interval for all patients was 3.53 years (standard deviation, SD = 1.04), with each patient visiting at least once. Comprehensive baseline and follow-up clinical data were obtained through face-to-face interviews. The patients were categorized into early-onset PD (EOPD) with an onset age of less than 50 years and late-onset PD (LOPD) with an onset age of over 50 years. Information on anti-PD medications was collected, and the levodopa equivalent daily dose (LEDD) was calculated, as previously described (17). Consistent with prior studies, PD patients were classified into three subtypes based on the symptomatology: tremor-dominant (TD) PD (mean tremor score/PIGD score ≥ 1.5), postural instability and gait disturbances (PIGD)-dominant PD (mean tremor score/PIGD score ≤ 1.0), and intermediate PD (1.0 < mean tremor score/PIGD score < 1.5) (18, 19). Motor symptom severity was assessed using the Unified Parkinson’s Disease Rating Scale (UPDRS) parts II and III and the Hoehn–Yahr (H–Y) stage. Specific motor features, including tremor (UPDRS items 20 and 21), rigidity (item 22), bradykinesia (items 23–26 and 31), and axial impairment (items 27–30), were further evaluated (20). Non-motor symptoms (NMSs) were assessed using the Non-Motor Symptoms Questionnaire (NMSQuest). Cognitive performance was evaluated using the Mini-Mental State Examination (MMSE) and Montreal Cognitive Assessment (MoCA). Emotional status was measured using the Hamilton Depression Rating Scale (HAM-D) and Hamilton Anxiety Rating Scale (HAM-A), while sleep quality was assessed using the Parkinson’s Disease Sleep Scale (PDSS). Neuropsychological assessments were conducted by highly trained specialist neurologists.

### 2.3 SNP genotyping

The GRIN2B phenotype of PD patients in our study was assessed using **iPLEX**^**®**^ reagents and the MassARRAY^®^ System (Sequenom Company) (21). Specifically, the PCR amplification primers and single-base extension primers for the SNP sites of GRIN2B were synthesized using genotyping tools and the MassARRAY Assay Design developed by the Sequenom Company, San Diego, CA, USA. The forward primer sequence was F:5’-ACGTTGGATGGCCTTCCCACCATTAATCTG-3′, and the reverse primer sequence was R:5’-ACGTTGGATGTGTGGAAGTAGCTGGGTATA-3′. Then, the peripheral blood samples were collected, and the DNA was extracted using a DNA extraction kit. The DNA was resuspended to a final concentration of 50 ng/μl and stored at −20°C. PCR amplification was performed by multiplex PCR in 384-well plates, with a total volume of 5 μL per reaction. The iPLEX assay was performed at the end of the PCR reaction. The PCR products were treated with shrimp alkaline phosphatase (SAP) to remove free deoxynucleotide triphosphates (dNTPs) from the system. Clean resin was used to desalt the iPLEX reaction products, optimizing MALDI-TOF mass spectrometric analysis. Then, the reaction products were put onto a 384-element SpectroCHIP bioarray. MassARRAY Workstation software was used to process and analyze the iPLEX SpectroCHIP bioarray. The genotypes of GRIN2B SNPs were determined through differences in the molecular weight of the PCR products.

### 2.4 Declaration of generative AI and AI-assisted technologies in the writing process

The authors declare that Generative AI was used in the creation of this manuscript. The authors used ChatGPT 4.0 to improve the readability and language of the manuscript. After using this tool, the authors reviewed and edited the content as needed and take full responsibility for the content of the published article.

### 2.5 Statistical analysis

All statistical analyses were conducted using IBM SPSS Statistics version 26.0. Categorical data were described using frequencies and percentages. To assess the representativeness of the study population, the Hardy–Weinberg equilibrium (HWE) was tested. The chi-squared (χ^2^) test was applied to compare the allele and genotype frequencies between the groups. Logistic regression analysis was performed to calculate the odds ratios (ORs) and their corresponding 95% confidence intervals (95% CIs). Both linear and logistic regression analyses were conducted to compare the baseline clinical characteristics across the different groups.

In longitudinal studies, linear mixed-effects models are often employed to analyze repeated measures, effectively managing missing data and accounting for individual variability. Consequently, these models were used to explore the association between the GRIN2B genotypes and the progression of motor and cognitive symptoms, as assessed through the UPDRS motor scores and MoCA scores. The fixed effects included the GRIN2B genotype, disease duration (as the timescale), and their interaction while adjusting for age, sex, and LEDD or education levels. The model incorporated random intercepts for each participant’s ID and random slopes for time to account for the correlations within repeated measures over time and among individuals.

## 3 Results

### 3.1 Demographics and clinical features of the participants

A total of 165 patients with PD and 154 healthy controls were enrolled in our study, comprising a total of 319 participants. The demographic and clinical data of these individuals are presented in Table 1. The distribution of sex, age, and years of education between the PD patients and healthy controls was found to be statistically similar. Among the PD patients, 43 (25.5%) individuals were classified as early-onset, with an average disease duration of 4.43 years (standard deviation, SD = 0.27) across all participants with PD. The mean follow-up duration was 3.53 years (SD = 0.08). At baseline, the median Hoehn–Yahr (H–Y) stage was 2.0, with an interquartile range of 1.5 to 2.5, and the average UPDRS III score was 21.55 (SD = 0.27). In addition, the mean MoCA score was 24.46 (SD = 0.34), and the MMSE score was 28.43 (SD = 0.18).

**Table 1:** Demographic data of the patients with sporadic PD and healthy controls.

Characteristic	PD (*n* = 165)	Control (*n* = 154)	*p*-value
Female sex, No. (%)	69 (41.82%)	73 (47.40%)	0.316
Age, mean (SD), y	68.96 (10.48)	67.22 (5.50)	0.371
Education, mean (SD), y	10.68 (3.78)	10.93 (3.78)	0.551
EOPD (>50 years), No. (%)	33 (20.00%)	—	—
Disease duration, mean (SD), y	4.43 (0.27)	—	—
Duration of follow-up, mean (SD), y	3.53 (0.08)	—	—
LEDD, mean (SD), mg	397.67 (19.42)	—	—
Hoehn–Yahr stage, median (IQR)	2 (1.5–2.5)	—	—
Right limb onset, No. (%)	81 (49.09%)	—	—
PD subtypes (PIGD/TD/Intermediate) No. (%)	79/61/25 (47.88%/36.97%/15.15%)	—	—
UPDRS II, mean (SD)	10.53 (0.45)	—	—
UPDRS III, mean (SD)	21.55 (0.92)	—	—
UPDRS motor subscores			
Tremors, mean (SD)	3.96 (0.26)	—	—
Rigidity, mean (SD)	3.60 (0.26)	—	—
Bradykinesia, mean (SD)	8.29 (0.45)	—	—
Axial impairment, mean (SD)	4.05 (0.22)	—	—
MoCA score, mean (SD)	24.46 (0.34)	—	—
Visuospatial/Executive, mean (SD)	3.36 (0.13)	—	—
Naming, mean (SD)	2.75 (0.04)	—	—
Attention, mean (SD)	5.29 (0.09)	—	—
Language, mean (SD)	2.57 (0.06)	—	—
Abstraction, mean (SD)	1.54 (0.06)	—	—
Recall, mean (SD)	3.04 (0.13)	—	—
Orientation, mean (SD)	5.90 (0.04)	—	—
MMSE score, mean (SD)	28.43 (0.18)	—	—
HAM-D score, mean (SD)	11.50 (0.75)	—	—
HAM-A score, mean (SD)	9.27 (0.59)	—	—
PDSS score, mean (SD)	119.22 (1.64)	—	—
NMSQuest score, mean (SD)	9.90 (0.40)	—	—

*p-values were calculated using linear regression or logistic regression. PD, Parkinson’s disease; EOPD, early-onset Parkinson’s disease; LEDD, levodopa equivalent daily dose; UPDRS, Unified Parkinson’s Disease Rating Scale; MMSE, mini-mental state examination; MoCA, Montreal Cognitive Assessment; HAM-D, Hamilton Depression Rating Scale; HAM-A, Hamilton Anxiety Rating Scale; NMSQuest, Non-Motor Symptoms Questionnaire; PDSS, Parkinson’s Disease Sleep Scale; NM-PD, non-GBA-mutated PD; PD, Parkinson’s disease; UPDRS, Unified Parkinson’s Disease Rating Scale.*

### 3.2 Correlation analysis between GRIN2B rs219882 and PD risk

The genotype and allele frequencies of the GRIN2B rs219882 polymorphism in both the PD and control groups are detailed in Table 2. The analysis for the HWE revealed no significant deviation in the allele frequencies within the PD group (*p* = 0.881) or the control group (*p* = 0.900), indicating that our study population was representative. Notably, the frequency of the GG genotype and the G allele was significantly higher in PD patients compared to the controls (OR = 2.27, 95% confidence interval (CI): 1.13–4.59, *P_genotype_* = 0.021; OR = 2.42, 95% CI: 1.72–3.40, *P_allele_* = 0.001). Furthermore, under a recessive model, PD patients with the GG + GT genotype showed a higher proportion compared to the healthy controls (OR = 1.98, 95% CI: 1.05–3.72, *p* = 0.032; adjusted OR = 1.99, 95% CI: 1.05–3.77), while no significant difference was observed between the two groups under a dominant model. These results suggest that the G allele of the GRIN2B rs219882 polymorphism is associated with PD susceptibility and that the G allele and GG + GT genotype may constitute risk factors for the development of PD.

**Table 2:** Genotype and allele frequency of the GRIN2B rs219882 (T > G) polymorphism among the patients with sporadic PD and controls in China.

	PD, No. (%)	Control, No. (%)	OR (95% CI)	*p*-value	Adjusted OR^*^ (95% CI)
HWE	0.881	0.900			
Genotype frequency					
GG	60 (36.4%)	44 (28.6%)	2.27 (1.13–4.59)	**0.021**	2.26 (1.11–4.59)
GT	87 (52.7%)	80 (51.9%)	1.25 (0.77–2.05)	0.075	1.23 (0.75–2.02)
TT	18 (10.9%)	30 (19.5%)	1		
Allele frequency					
G	207 (62.7%)	168 (54.5%)	2.42 (1.72–3.40)	**0.001**	2.41 (1.71–3.40)
T	123 (37.3%)	165 (45.5%)	1		
Dominant model					
GG	60 (36.4%)	44 (28.6%)	1.43 (0.89–2.23)	0.138	1.40 (0.87–2.26)
GT + TT	105 (63.6%)	110 (71.4%)	1		
Recessive model					
GG + GT	147 (89.1%)	124 (80.5%)	1.98 (1.05–3.72)	**0.032**	1.99 (1.05–3.77)
TT	18 (10.9%)	30 (19.5%)	1		

^*^OR was adjusted for age and sex. *p*-values were calculated using the χ^2^ test. The values in bold represent the statistically significant differences (*p* < 0.05). PD, Parkinson’s disease; HWE, Hardy–Weinberg equilibrium; and N-methyl-D-aspartate receptor 2B (GRIN2B).

### 3.3 Association analysis between GRIN2B rs219882 and the age onset of PD

We conducted a stratified analysis based on the age of onset of PD, as presented in Table 3. In the LOPD subgroup, the frequency of the G allele was significantly higher in PD patients compared to the controls (OR = 1.55, 95%CI: 1.09–2.20, *p* = 0.014). In addition, under a dominant model, the frequency of the GG genotype was more prevalent in the PD group than in the controls (OR = 1.70, 95% CI: 1.01–2.85, *p* = 0.045). Most notably, a higher proportion of the GG + GT genotype was observed in the PD group compared to the healthy controls in a recessive model (OR = 2.11, 95% CI: 1.04–4.25, *p* = 0.035). Collectively, these findings suggest that the G allele and the GG + GT genotype may be associated with an increased susceptibility to PD, particularly in the context of LOPD.

**Table 3:** Distributions of the genotype and allele frequencies of GRIN2B rs219882 (T > G) in the sporadic PD and control groups stratified by sex or age at onset.

		PD, No. (%)	Control, No. (%)	OR (95% CI)	*p*-value
EOPD vs. Control	Dominant model				
	GG	8 (24.2%)	8 (33.3%)	0.64 (0.20–2.05)	0.451
	GT + TT	25 (75.8%)	16 (66.7%)	1	
	Recessive model				
	GG + GT	29 (87.9%)	20 (83.3%)	1.45 (0.32–6.49)	0.919
	TT	4 (12.1%)	4 (16.7%)	1	
	Allele frequency				
	G	37 (56.1%)	28 (66.7%)	0.91 (0.43–1.93)	0.809
	T	29 (43.9%)	20 (47.6%)	1	
LOPD vs. Control	Dominant model				
	GG	52 (39.4%)	36 (27.7%)	1.70 (1.01–2.85)	**0.045**
	GT + TT	80 (60.6%)	94 (72.3%)	1	
	Recessive model				
	GG + GT	118 (89.1%)	104 (80.0%)	2.11 (1.04–4.25)	**0.035**
	TT	14 (10.9%)	26 (20.0%)	1	
	Allele frequency				
	G	170 (64.4%)	140 (53.8%)	1.55 (1.09–2.20)	**0.014**
	T	94 (35.6%)	120 (46.2%)	1	

*p*-values were calculated using the χ^2^ test. The values in bold represent the statistically significant differences (*p* < 0.05). PD, Parkinson’s disease; EOPD, early-onset Parkinson’s disease; LOPD, late-onset Parkinson’s disease; N-methyl-D-aspartate receptor 2B (GRIN2B).

### 3.4 Effect of GRIN2B rs219882 on motor or cognitive decline

We conducted a detailed longitudinal cohort study to examine whether the GRIN2B rs219882 genotype frequency influences the progression of motor and cognitive functions in PD. We performed a detailed longitudinal cohort study of 89 PD patients and the baseline clinical features of 165 PD patients are summarized in Table 3. The mean follow-up time was 3.53 (1.06) years for the GG + GT carriers and 3.51 (0.88) years for the TT carriers. At baseline, the distributions of age, sex, disease duration, education, LEDD, H–Y stages, and cognitive impairment were similar between the two groups. Interestingly, the individuals with the GG + GT genotype exhibited higher UPDRS motor scores (*p* = 0.008), particularly in rigidity (*p* = 0.003), bradykinesia (*p* = 0.012), and axial impairment (*p* = 0.045), with no significant difference observed in the tremor scores.

**Figure 2:**
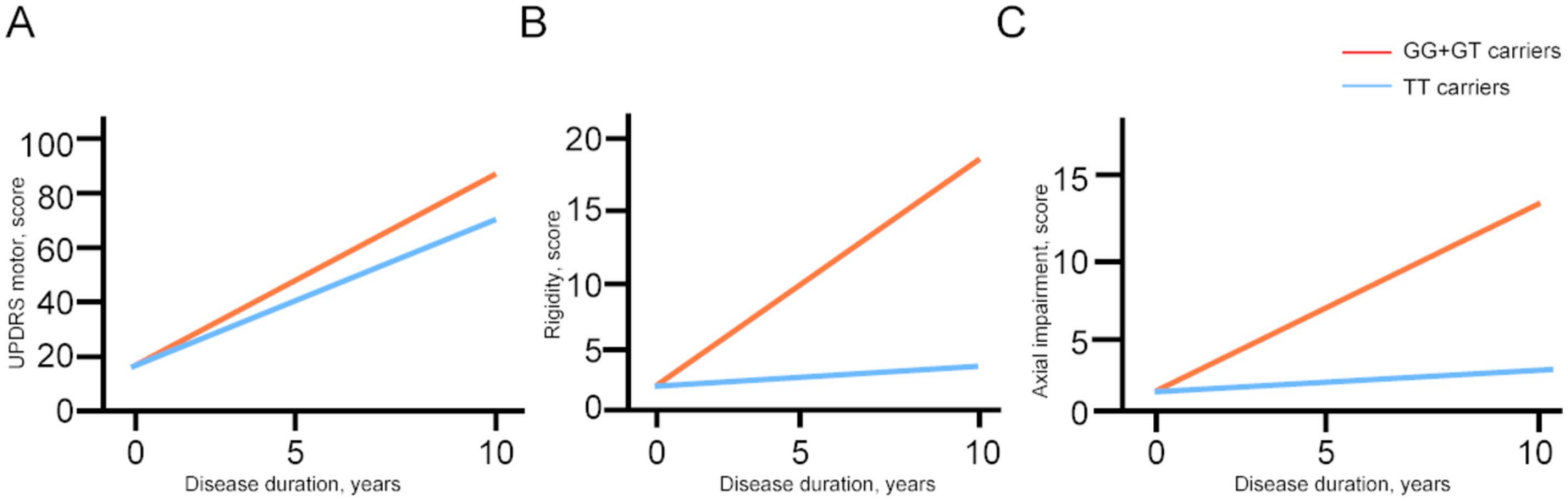
The longitudinal trajectories of the mean UPDRS motor scores and subparts among the GRIN2B genotypes. UPDRS, Unified Parkinson’s Disease Rating Scale; N-methyl-D-aspartate receptor 2B (GRIN2B). Rigidity scores, bradykinesia scores, and axial impairment scores were evaluated using the item 22, items 23–26, 31, items 27–30 respectively.

Linear mixed-effects models were used to examine the correlation between the GRIN2B genotype and the progression rate of UPDRS motor scores and its subparts, adjusting for sex, age at baseline, and LEDD at follow-up (Table 4; Figure 2). The interaction with time revealed the influence of the genotype status on the slope (annual change in the scores of the UPDRS motor and its subparts). The estimated progression rate (standard error, SE) of the UPDRS motor score was 6.19 (2.56) points/year for PD patients with the GG genotype, 5.99 (2.44) points/year for those with the GT genotype, and 5.42 (2.47) points/year for those with the TT genotype. Notably, the progression rate for the patients with the GG + GT genotype [6.10 (2.54) points/year; *p* = 0.022] was significantly faster than that of those with the TT genotype. The analysis of the subparts of the UPDRS motor scores revealed that the SE rate of change in the rigidity and axial impairment scores was significantly faster in the GG + GT carriers compared to the TT carriers [1.83 (0.58) points/year; *P_rigidity_* = 0.003; 1.20 (0.55) points/year; *P_axial impairment_* = 0.033]. These findings suggest that the GG + GT genotype is associated with a faster motor decline, particularly in terms of rigidity and axial impairment.

**Table 4:** Association between GRIN2B rs219882 and motor progression among the groups.

Slope, points/year					
	GG	GT	TT	GG + GT vs. TT	
Characteristic	B (SE)	B (SE)	B (SE)	B (SE)	*p*-value
Total MDS-UPDRS III Score	6.19 (2.56)	5.99 (2.44)	5.42 (2.47)	0.68 (2.44)	**0.022**
Tremors	0.62 (0.61)	0.51 (0.55)	0.36 (0.62)	0.15 (0.56)	0.790
Rigidity	2.08 (0.6)	1.72 (0.54)	0.08 (0.63)	1.75 (0.55)	**0.003**
Bradykinesia	2.61 (1.11)	2.68 (0.99)	0.77 (1.06)	1.95 (0.88)	0.104
Axial impairment	1.27 (0.52)	0.55 (0.60)	0.16 (0.57)	1.04 (0.49)	**0.033**

Interaction with time (slope, points/year). Model adjusted for age, sex, and LEDD at follow-up. The interaction with time indicates the effect of the GRIN2B genotype status on the slope (annual change in the UPDRS motor scores and subscores). The values in bold represent the statistically significant differences (*p* < 0.05). UPDRS, Unified Parkinson’s Disease Rating Scale; N-methyl-D-aspartate receptor 2B (GRIN2B).

To further ascertain the impact of the GRIN2B rs219882 genotype on cognitive decline, we employed a linear mixed-effects model to examine the rate of change in the MoCA scores and its seven subscale scores, adjusting for age, sex, and years of formal education at baseline (Table 5). Notably, the rate of deterioration in the recall score for the patients with the GG genotype was 0.58 points/year higher than that for the carriers of the TT genotype (*p* = 0.030). However, no significant differences were observed in the progression rates of the MoCA and its subscale scores.

**Table 5:** Association between GRIN2B rs219882 and cognitive progression among the groups.

Slope, points/year	Genotype					
	GG vs. TT		GT vs. TT		GG + GT vs. TT	
Characteristic	B (SE)	*p*-value	B (SE)	*p*-value	B (SE)	*p*-value
MoCA						
Interaction with time	1.53 (0.85)	0.090	0.70 (0.78)	0.379	1.03 (0.86)	0.246
Visuospatial/executive						
Interaction with time	0.58 (0.33)	0.094	0.44 (0.30)	0.172	0.48 (0.31)	0.134
Naming						
Interaction with time	0.02 (0.11)	0.881	0.08 (0.12)	0.465	0.02 (0.11)	0.888
Attention						
Interaction with time	0.15 (0.19)	0.434	0.05 (0.17)	0.755	0.01 (0.18)	0.973
Language						
Interaction with time	0.09 (0.14)	0.518	0.06 (0.13)	0.724	0.06 (0.13)	0.624
Abstraction						
Interaction with time	0.11 (0.12)	0.366	0.02 (0.11)	0.826	0.05 (0.11)	0.664
Recall						
Interaction with time	0.58 (0.27)	0.030	0.39 (0.25)	0.115	0.44 (0.24)	0.074
Orientation						
Interaction with time	0.02 (0.14)	0.912	0.11 (0.12)	0.362	0.04 (0.15)	0.779

Model adjusted for age, sex, and education at follow-up, while the interaction with time indicates the effect of the GRIN2B genotype status on the slope (annual change in the MoCA scores and subscores). The values in bold represent the statistically significant differences (*p* < 0.05). Abbreviations: MoCA, Montreal Cognitive Assessment; N-methyl-D-aspartate receptor 2B (GRIN2B).

## 4 Discussion

To the best of our knowledge, our research is the first to highlight the relationship between GRIN2B SNPs and the motor progression of PD within a longitudinal Chinese cohort. Our study revealed that the GRIN2B rs219882 G allele is associated with PD susceptibility, particularly in LOPD. Notably, the individuals with the GRIN2B GG + GT genotype exhibited a more rapid motor decline, especially in terms of rigidity and axial impairment, compared to the TT genotype carriers.

We found that the risk of PD in the G allele carriers was 2.4 times higher than that in the non-carriers. The GRIN2B rs219882T > G variant, an intron variant located within gene regions, likely contributes to transcriptional dysfunction (22). Elevated GRIN2B levels are implicated in PD pathogenesis through TNF-*α*-associated neuroinflammation, which may account for our findings (23). Notably, the G allele frequency in the healthy controls (0.455) in our study is consistent with that of East Asian populations in the dbSNP database (0.425), suggesting strong statistical power for this SNP in our cohort. Furthermore, our study demonstrated that the G allele significantly influenced the age of PD onset. The rs219882 variant was initially identified in a genome-wide association study (GWAS) of patients with sporadic LOPD (22). Our findings are consistent with this research, showing a higher frequency of the G allele in LOPD individuals compared to the healthy controls. LOPD is characterized by more severe dopaminergic damage and motor impairment, as well as a higher rate of progression compared to EOPD, due to distinct underlying molecular mechanisms (24, 25). Our results suggest that the pathogenic mechanisms of GRIN2B variations may share similarities with those of LOPD.

The UPDRS motor score, a standardized measure for evaluating motor impairment severity, is well-established for its reliability and validity (26, 27) and is frequently used in clinical research (28). However, few studies have utilized the UPDRS to assess the rate of motor progression. In our PD cohort, the GG + GT carriers experienced a faster rate of motor decline compared to the TT carriers, as estimated by the UPDRS scores. Moreover, we found that the GG carriers had a more rapid motor decline than the GT carriers, suggesting a correlation between the rate of motor decline and the severity of the GRIN2B variant. This suggests that GRIN2B variant severity is likely involved in GRIN2B function regulation, contributing to PD development and progression. Consistent with our findings, previous animal studies have confirmed that GRIN2B dysfunction impacts the progression of motor dysfunction in PD (10, 23, 29). As the GRIN2B G allele is associated with a faster rate of motor progression, we further explored the potential association between the GRIN2B genotype and specific motor features. We found that the GG + GT carriers had faster progression rates in the rigidity and axial impairment scores than the TT carriers. NMDA antagonists have been shown to alleviate rigidity in a PD mouse model (30, 31). Interestingly, we also found that the rate of bradykinesia decline was faster in the GT carriers than in the TT carriers. It has been reported that co-administration of levodopa with NMDA antagonists can potentiate the effect of levodopa and reduce levodopa-induced dyskinesias (30, 32). Our results indicate that GRIN2B variations play a pivotal role in the rigidity or dyskinesia symptoms of PD.

An intriguing finding was that the GG carriers lost 0.58 points in recall per year, which was significantly faster than the TT carriers. GRIN2B is crucial for synaptic and cognitive function in an *α*-synuclein-induced mouse model, possibly due to an imbalance in calcium homeostasis (10, 33). However, the difference in total MoCA scores between the GG and TT carriers was not significant. Further studies are needed to elucidate the association between GRIN2B and cognitive progression.

The strengths of this study include the following: (1) demonstrating a novel association between the GRIN2B rs219882 G allele and PD susceptibility and age of onset in a Chinese population and (2) being the first to discover that GRIN2B GG + GT carriers experience rapid motor progression, characterized by more severe rigidity and axial impairment, in a longitudinal study of PD patients from China. Our research may provide theoretical support for predicting the rapid progression of motor and cognitive impairments in patients with LOPD. Consequently, patients with LOPD could potentially predict the progression of their motor and cognitive functions by detecting GRIN2B gene polymorphisms, thereby guiding their clinical treatment. However, there are limitations to this study. Complete genotype testing is required to mitigate potential confounding factors from prevalent genes associated with PD, such as *LRRK2*, *APOE*, and *PRKN*. In addition, this retrospective study had missing data and inhomogeneous follow-up times. To address these issues, we performed linear mixed-effects models to account for potential confounders and handle the missing data. Finally, our study was a single-center follow-up research with a relatively small sample size. Therefore, it is necessary to conduct multi-center studies with larger populations to validate our findings.

## 5 Conclusion

Our study represents a pioneering effort in examining the role of GRIN2B in the prevalence and motor and cognitive functional progression of PD within the Chinese population. Our findings reveal that PD patients with GG + GT genotypes experience a more rapid decline in motor function, particularly in the progression of rigidity and axial impairment. Notably, GG genotype carriers exhibit a more rapid decline in recall function. These insights are of significant importance for understanding the impact of GRIN2B variants on the pattern of motor deterioration in PD. They have the potential to help clinicians make more accurate prognoses and help inform treatment strategies.

## Data Availability

The original contributions presented in the study are included in the article, further inquiries can be directed to the corresponding author/s.

## References

[1] Bloem BR Okun MS Klein C. Parkinson's disease. Lancet. (2021) 397:2284–303. doi: 10.1016/S0140-6736(21)00218-X 33848468

[2] Schapira AHV Chaudhuri KR Jenner P. Non-motor features of Parkinson disease. Nat Rev Neurosci. (2017) 18:435–50. doi: 10.1038/nrn.2017.62 28592904

[3] Pang SY Ho PW Liu HF Leung CT Li L Chang EES. The interplay of aging, genetics and environmental factors in the pathogenesis of Parkinson's disease. Transl Neurodegener. (2019) 8:23. doi: 10.1186/s40035-019-0165-9, PMID: 31428316 PMC6696688

[4] Rizig M Bandres-Ciga S Makarious MB Ojo OO Crea PW Abiodun OV. Identification of genetic risk loci and causal insights associated with Parkinson's disease in African and African admixed populations: a genome-wide association study. Lancet Neurol. (2023) 22:1015–25. doi: 10.1016/S1474-4422(23)00283-1, PMID: 37633302 PMC10593199

[5] Song R Zhang J Perszyk RE Camp CR Tang W Kannan V. Differential responses of disease-related GRIN variants located in pore-forming M2 domain of N-methyl-D-aspartate receptor to FDA-approved inhibitors. J Neurochem. (2023) 168:3936–49. doi: 10.1111/jnc.15942 37649269 PMC10902181

[6] Iacobucci GJ Popescu GK. NMDA receptors: linking physiological output to biophysical operation. Nat Rev Neurosci. (2017) 18:236–49. doi: 10.1038/nrn.2017.24, PMID: 28303017 PMC5640446

[7] Endele S Rosenberger G Geider K Popp B Tamer C Stefanova I. Mutations in GRIN2A and GRIN2B encoding regulatory subunits of NMDA receptors cause variable neurodevelopmental phenotypes. Nat Genet. (2010) 42:1021–6. doi: 10.1038/ng.677, PMID: 20890276

[8] Carvill GL Regan BM Yendle SC O'Roak BJ Lozovaya N Bruneau N. GRIN2A mutations cause epilepsy-aphasia spectrum disorders. Nat Genet. (2013) 45:1073–6. doi: 10.1038/ng.2727, PMID: 23933818 PMC3868952

[9] Paoletti P Neyton J. NMDA receptor subunits: function and pharmacology. Curr Opin Pharmacol. (2007) 7:39–47. doi: 10.1016/j.coph.2006.08.011 17088105

[10] Ferreira DG Temido-Ferreira M Vicente Miranda H Batalha VL Coelho JE Szegö ÉM. α-synuclein interacts with PrPC to induce cognitive impairment through mGluR5 and NMDAR2B. Nat Neurosci. (2017) 20:1569–79. doi: 10.1038/nn.4648, PMID: 28945221

[11] Wu SL Wang WF Shyu HY Ho YJ Shieh JC Fu YP. Association analysis of GRIN1 and GRIN2B polymorphisms and Parkinson's disease in a hospital-based case-control study. Neurosci Lett. (2010) 478:61–5. doi: 10.1016/j.neulet.2010.04.063, PMID: 20438806

[12] Zainal Abidin S Tan EL Chan SC Jaafar A Lee AX Abd Hamid MHN. DRD and GRIN2B polymorphisms and their association with the development of impulse control behaviour among Malaysian Parkinson's disease patients. BMC Neurol. (2015) 15:59. doi: 10.1186/s12883-015-0316-2, PMID: 25896831 PMC4417293

[13] Tsai SJ Liu HC Liu TY Cheng CY Hong CJ. Association analysis for genetic variants of the NMDA receptor 2b subunit (GRIN2B) and Parkinson's disease. J Neural Transm (Vienna). (2002) 109:483–8. doi: 10.1007/s007020200039 11956967

[14] Li J Yi M Li B Yin S Zhang Y Huang Z. Polymorphism of neurodegeneration-related genes associated with Parkinson's disease risk. Neurol Sci. (2022) 43:5301–12. doi: 10.1007/s10072-022-06192-8, PMID: 35695987

[15] Krishnamoorthy S Rajan R Banerjee M Kumar H Sarma G Krishnan S. Dopamine D3 receptor Ser9Gly variant is associated with impulse control disorders in Parkinson's disease patients. Parkinsonism Relat Disord. (2016) 30:13–7. doi: 10.1016/j.parkreldis.2016.06.005, PMID: 27325396

[16] Postuma RB Berg D Stern M Poewe W Olanow CW Oertel W. MDS clinical diagnostic criteria for Parkinson's disease. Mov Disord. (2015) 30:1591–601. doi: 10.1002/mds.26424 26474316

[17] Jost ST Kaldenbach MA Antonini A Martinez-Martin P Timmermann L Odin P. Levodopa Dose Equivalency in Parkinson's Disease: Updated Systematic Review and Proposals. Mov Disord. (2023) 38:1236–52. doi: 10.1002/mds.29410, PMID: 37147135

[18] Davis MY Johnson CO Leverenz JB Weintraub D Trojanowski JQ Chen-Plotkin A. Association of GBA Mutations and the E326K Polymorphism With Motor and Cognitive Progression in Parkinson Disease. JAMA Neurol. (2016) 73:1217–24. doi: 10.1001/jamaneurol.2016.2245, PMID: 27571329 PMC5056861

[19] Stebbins GT Goetz CG Burn DJ Jankovic J Khoo TK Tilley BC. How to identify tremor dominant and postural instability/gait difficulty groups with the movement disorder society unified Parkinson's disease rating scale: comparison with the unified Parkinson's disease rating scale. Mov Disord. (2013) 28:668–70. doi: 10.1002/mds.25383, PMID: 23408503

[20] Arpin DJ Mitchell T Archer DB Burciu RG Chu WT Gao H. Diffusion Magnetic Resonance Imaging Detects Progression in Parkinson's Disease: A Placebo-Controlled Trial of Rasagiline. Mov Disord. (2022) 37:325–33. doi: 10.1002/mds.28838, PMID: 34724257 PMC9019575

[21] Oeth P. Beaulieu M. Park C. Kosman D. del Mistro G. van den Boom D.., iPLEX™ assay: increased plexing efficiency and flexibility for MassARRAY system through single base primer extension with mass-modified terminators. (2005). Available at: https://www.researchgate.net/publication/290120569 (Accessed September 27, 2007).

[22] Hamza TH Zabetian CP Tenesa A Laederach A Montimurro J Yearout D. Common genetic variation in the HLA region is associated with late-onset sporadic Parkinson's disease. Nat Genet. (2010) 42:781–5. doi: 10.1038/ng.642, PMID: 20711177 PMC2930111

[23] Yan J Liu A Fan H Qiao L Wu J Shen M. Simvastatin Improves Behavioral Disorders and Hippocampal Inflammatory Reaction by NMDA-Mediated Anti-inflammatory Function in MPTP-Treated Mice. Cell Mol Neurobiol. (2020) 40:1155–64. doi: 10.1007/s10571-020-00804-7, PMID: 32016638 PMC11448790

[24] Schirinzi T di Lazzaro G Sancesario GM Summa S Petrucci S Colona VL. Young-onset and late-onset Parkinson's disease exhibit a different profile of fluid biomarkers and clinical features. Neurobiol Aging. (2020) 90:119–24. doi: 10.1016/j.neurobiolaging.2020.02.012, PMID: 32169356

[25] Pagano G Ferrara N Brooks DJ Pavese N. Age at onset and Parkinson disease phenotype. Neurology. (2016) 86:1400–7. doi: 10.1212/WNL.0000000000002461, PMID: 26865518 PMC4831034

[26] Guo Y Goetz CG Stebbins GT Mestre TA Luo S. Using Movement Disorder Society Unified Parkinson's Disease Rating Scale Parts 2 and 3 Simultaneously: Combining the Patient Voice with Clinician Ratings. Mov Disord. (2023) 38:453–63. doi: 10.1002/mds.29308, PMID: 36621935 PMC10033355

[27] Elfil M Bahbah EI Attia MM Eldokmak M Koo BB. Impact of Obstructive Sleep Apnea on Cognitive and Motor Functions in Parkinson's Disease. Mov Disord. (2021) 36:570–80. doi: 10.1002/mds.28412, PMID: 33296545

[28] Choudhury P Zhang N Adler CH Chen K Belden C Driver-Dunckley E. Longitudinal motor decline in dementia with Lewy bodies, Parkinson disease dementia, and Alzheimer's dementia in a community autopsy cohort. Alzheimers Dement. (2023) 19:4377–87. doi: 10.1002/alz.13357, PMID: 37422286 PMC10592344

[29] Canerina-Amaro A Pereda D Diaz M Rodriguez-Barreto D Casañas-Sánchez V Heffer M. Differential Aggregation and Phosphorylation of Alpha Synuclein in Membrane Compartments Associated With Parkinson Disease. Front Neurosci. (2019) 13:382. doi: 10.3389/fnins.2019.00382, PMID: 31068782 PMC6491821

[30] Johnson KA Conn PJ Niswender CM. Glutamate receptors as therapeutic targets for Parkinson's disease. CNS Neurol Disord Drug Targets. (2009) 8:475–91. doi: 10.2174/187152709789824606, PMID: 19702565 PMC3005251

[31] Karcz-Kubicha M Lorenz B Danysz W. GlycineB antagonists and partial agonists in rodent models of Parkinson's disease--comparison with uncompetitive N-methyl-D-aspartate receptor antagonist. Neuropharmacology. (1999) 38:109–19. doi: 10.1016/S0028-3908(98)00165-8, PMID: 10193902

[32] Marin C Papa S Engber TM Bonastre M Tolosa E Chase TN. MK-801 prevents levodopa-induced motor response alterations in parkinsonian rats. Brain Res. (1996) 736:202–5. doi: 10.1016/0006-8993(96)00693-2, PMID: 8930325

[33] Zhang X-H Liu SS Yi F Zhuo M Li BM. Delay-dependent impairment of spatial working memory with inhibition of NR2B-containing NMDA receptors in hippocampal CA1 region of rats. Mol Brain. (2013) 6:13. doi: 10.1186/1756-6606-6-13, PMID: 23497405 PMC3616959

